# Polypore mushroom mycelia as an adjunct to COVID-19 vaccination: a randomized clinical trial

**DOI:** 10.1186/s12865-026-00809-9

**Published:** 2026-01-31

**Authors:** Gordon Saxe, Christine N. Smith, Shahrokh Golshan, Tatyana Shekhtman, Zolton J. Bair, Chase Beathard, Renee A. Davis, Lauray MacElhern, Andrew Shubov, Daniel Slater, Lan K. Kao, Phoebe Senowitz, Stephen Wilson

**Affiliations:** 1 Department of Family Medicine, University of California San Diego, San Diego, CA USA; 2 Krupp Center for Integrative Research, Centers for Integrative Health, University of California San Diego, San Diego, CA USA; 3 Veterans Affairs San Diego Healthcare System, San Diego, CA USA; 4 Department of Psychiatry, University of California San Diego, San Diego, CA USA; 5 Center for the Neurobiology of Learning and Memory, University of California Irvine, Irvine, CA USA; 6 Department of Research and Development, Fungi Perfecti, LLC, Washington, Olympia USA; 7 Department of Civil and Environmental Engineering, University of Washington, Seattle, WA USA; 8 Department of Medicine, Center for East-West Medicine, University of California Los Angeles, Los Angeles, CA USA; 9 Botnar Institute of Immune Engineering, Basel, Switzerland

**Keywords:** Vaccine, COVID, Bird flu, Fungi, Mushroom, Mycelium, Side effects, Antibodies, Adjunct, SARS-CoV-2

## Abstract

**Background:**

Use of fungal mycelium as a vaccination adjunct may constitute a novel antiviral strategy to address newly emerging viruses. We evaluated safety and feasibility of a fungal mycelium-based natural product (*Fomitopsis officinalis* and *Trametes versicolor*, FoTv) as an adjunct to human COVID-19 vaccination, as well as its impact on vaccine side-effects and anti-SARS-CoV-2 antibodies (Abs).

**Methods:**

Randomized, double-blind, placebo-controlled clinical trial involving adjunctive treatment with FoTv or visually-identical Placebo (dosage: eight 500-mg capsules TID orally for four days) in combination with COVID-19 vaccination. Main outcomes included: Safety (adverse events, renal and hepatic function [Days 1–14]); Feasibility (completion rate and treatment adherence); Side-effects (number and severity, self-reported on days 1 [vaccination] to 5); and anti-SARS-CoV-2 Ab levels (receptor-binding domain and Spike, collected from blood drawn on days 1, 3, 14, and 28/42, and at 6 months).

**Results:**

Ninety participants receiving COVID-19 vaccination were randomized to either FoTv (*N* = 52) or Placebo (*N* = 38) groups. There were no adverse events and the groups had overlapping 95% confidence intervals for the percentage of participants transitioning from normal to abnormal renal/hepatic function when comparing Days 1 and 14. All participants (100%) completed the study and treatment adherence was greater than 95%. Participants with detectable anti-SARS-CoV-2 Abs (from prior COVID antigen exposure) were classified as “COVID-Exposed” and those with undetectable anti-SARS-CoV-2 Abs as “COVID-Naive.” FoTv, versus Placebo, significantly reduced side-effects in COVID-Naive individuals, specifically on days 3 and 5, but not in COVID-Exposed individuals. In the COVID-Naive FoTv group, Ab responses were preserved across 6 months (and possibly increased), an effect not observed among other groups.

**Conclusions:**

After COVID-19 vaccination, adjunctive FoTv was safe, feasible, and reduced vaccine side-effects without compromising (and possibly increasing) Ab levels up to 6 months in participants without previous SARS-CoV-2 exposure. Use of fungal mycelia was successfully tested as a unique approach to prevent a novel pandemic virus (SARS-CoV-2), with potential application to H5N1/Bird Flu and other emerging viruses.

**Trial registration:**

Trial registered on ClinicalTrials.gov NCT04951336 on June 30, 2021.

**Supplementary Information:**

The online version contains supplementary material available at 10.1186/s12865-026-00809-9.

## Introduction

### Lessons from the COVID-19 pandemic

As we take stock of the societal response to COVID-19 and plan for future pandemics, it has become clear that the medical community needs to identify successful strategies to minimize morbidity, mortality, and global disruption that could result both from newly evolving strains of SARS-CoV-2 as well as from other viruses or diseases (e.g., bird flu, monkey pox, Zika virus, Hantavirus, dengue fever, etc.) that could broadly threaten human health. Various safe and effective strategies, encompassing primary prevention, vaccination, antiviral medications, and diet/lifestyle modifications, are urgently needed to protect against existing and emerging strains of infectious diseases.

Strategies that incorporate vaccines/antiviral medication as the first lines of defense will likely be relied upon. Although vaccination has a long history of successful use in prevention of many viral and other illnesses, concerns have been raised about durability and cross-protective immunity conferred by mRNA and other forms of vaccination against SARS-CoV-2, as well as about vaccine-associated side effects. Unfortunately, antiviral medications also cannot be relied upon as a primary strategy because they: (a) may be in short supply/inaccessible to many individuals (for financial reasons or due to problems with distribution); (b) may encounter drug resistance because of overuse (e.g., prophylactic administration to livestock); and (c) are only likely to be effective if taken during a limited time window during the disease course (shortly after exposure or, at most, immediately after symptom onset).

In addition to COVID-19, other emerging infectious diseases also warrant close attention. Among these, highly-pathogenic avian influenza A (HPAI) is of particular and growing concern. HPAI, caused by a viral subtype of H5N1, has led to a serious form of bird flu with symptoms in humans ranging from mild (e.g., conjunctivitis, flu-like symptoms) to severe/fatal (e.g., respiratory failure). Between 2003 and 2024, 463/896 cases of HPAI due to H5N1 were fatal (52% case-fatality rate per reports from 24 countries) [1]. According to the Centers for Disease Control and Prevention (CDC), a new H5N1 clade (2.3.4.4b) was first detected in animals in 2021, with the first human case reported in January 2022. Although these cases have generally resulted in mild illness, this clade has been detected in over 500 avian and mammalian species globally and, more recently, in poultry, cattle, swine, and dairy products. The increasing cross-species transmissibility (avian-mammal, mammal-mammal, avian-human, and mammal-human) and broadening host range of this clade raise concern that sustained human-human transmission of this virus could soon develop [2]. This, in tandem with continuing outbreaks of this clade in animals and the high case-fatality rate of previous clades, has led to worry that a global pandemic virus more lethal and disruptive than SARS-CoV-2 could emerge.

### Vaccination as a strategy to address newly emerging or potentially pandemic infectious diseases

Vaccination was epidemiologically impactful in resolving the COVID-19 pandemic. This was possible because the virus was identified early and new technology (i.e., mRNA vaccines) was quickly and specifically designed to exploit points of viral vulnerability (e.g., Spike protein). Yet, there were shortcomings to this strategy. First, concerns persisted regarding side effect frequency and severity. Second, vaccine efficacy was often suboptimal, particularly in immunocompromised individuals, and tended to wane over time. Third, new strains of SARS-CoV-2 continued to emerge, resulting in further reductions in vaccine efficacy and durability, and need for more frequent boosters.

Not surprisingly, a significant portion of the population expressed vaccine hesitancy. According to a CDC report on a December 2020 survey coinciding with initial rollout of COVID-19 vaccines, approximately 30% of people not intending to be vaccinated identified “concern about side effects and safety” as their primary reason [3]. Later, a similar proportion identified concern about vaccine side effects as their reason for not receiving a booster [4]. Vaccine-related side effects are commonly mild and short-term, but are occasionally serious or longer-term [5]. Frequency of side effects, particularly common short-term ones, may be related to inflammation and heightened immunoreactivity to vaccine adjuvants [6] and may worsen with re-exposure [7, 8]. Because reactogenic side effects impede public health goals of widespread vaccination, an important challenge is to identify adjunctive agents/approaches that reduce excessive reactogenicity without compromising immunogenicity.

Vaccine immunogenicity is influenced by various factors related to immunological status, including diabetes [9], chronic renal failure [10], chronic liver disease [10, 11], steroid use [12], prior infection [13], age [14, 15], gender [16], nutritional status [10, 17], obesity [18], gut microbial balance [19], antibiotic use [20], and prior vaccination/infection [21]. Post-vaccine levels of SARS-CoV-2 anti-Spike protein antibodies (Abs) are correlated with and may serve as surrogates for protection against infection [22]. Unfortunately, levels of neutralizing Abs are insufficient to afford robust protection to many immunocompromised individuals [23, 24]. Further, Ab levels wane over time, marked by increasing rates of COVID-19 infection within 2–6 months post-vaccination [25, 26]. 

During the pandemic, extensive use of vaccines to prevent infection by SARS-CoV-2 (a virus to which the population was initially immunologically naive) revealed widespread vaccine hesitancy (particularly related to side effects concerns), and safety and efficacy concerns. Such concerns need to be addressed because vaccines will likely serve as a first defense to address new SARS-CoV-2 variants or other emerging viruses with pandemic potential. The use of fungal mycelium, in conjunction with immunization, may offer a novel strategy to reduce adverse side effects without compromising (or perhaps even enhancing) immunogenicity, thereby enhancing vaccine acceptance and efficacy.

### Vaccine enhancement with agaricomycete fungi

Agaricomycete fungi, consisting of mycelium (root-like network) and fruit bodies, have been studied as vaccine adjuvants in animal models and used in veterinary care; examples include *Phellinus linteus* (Pl) [27] mycelium extract, turkey tail *(Trametes versicolor*, Tv) polysaccharides including polysaccharide-K (PSK) [28], and *Pleurotus ostreatus* lectin [29]. Mice receiving intranasal coadministration of influenza A vaccine and an adjuvant of Pl mycelium extract and then challenged with variant H5N1 viruses, showed significantly reduced viral titers and greater survival rates compared to non-adjuvanted controls [27]. More recently, coadministration of SARS-CoV-2 vaccine (BNT162b2; Pfizer-BioNTech) and an adjuvant Tv mycelial extract (in combination with an extract of the Chinese herb *Astragalus membranaceus)* in mice, improved antigen (Ag)-binding efficacy against Spike proteins of SARS-CoV-2 [30]. In both studies, mycelial adjuvants to vaccination not only demonstrated efficacy against the targeted virus, but also induced cross-protection against variant strains of each respective virus. These findings suggest that agaricomycete fungal preparations may have appeal as candidate vaccine adjuvants/adjuncts.

Tv, a polypore fungus, has also demonstrated immunomodulatory [31, 32], anti-cancer [33–35], and antiviral activities [36, 37]. Tv mycelium modulated in vitro immune activity by increasing CD69 expression on human monocytes and lymphocytes, while the fermented substrate elicited more pronounced, dose-dependent increases in pro-inflammatory, anti-inflammatory, and antiviral cytokines [31]. Tv has been investigated as an adjunct to either chemotherapy or radiotherapy in at least thirteen clinical trials for cancer, with many of these trials focusing on mycelium-derived PSK, and demonstrating potential survival benefits across various cancer types including breast, colorectal, gastric, and esophagus [35]. Moreover, in breast cancer patients undergoing post-chemotherapy radiation, Tv increased lymphocyte and CD8 + T-cell counts, as well as tumoricidal activity of natural killer cells [34]. Tv has consistently demonstrated anti-influenza activity, and in a comparative study of multiple mushroom species, Tv mycelium emerged as the most promising candidate for anti-influenza and anti-herpetic applications, owing to its potent therapeutic efficacy and low toxicity [38, 39]. 

Another polypore, agarikon (*Fomitopsis officinalis* syn. *Laricifomes officinalis*, Fo), has been evaluated for the medicinal potential [40] of both its mycelium and fruit bodies, demonstrating wide-ranging antiviral, antibacterial, anti-cancer, and anti-inflammatory effects [41] suggestive of immunological activity [42]. Fo exhibited promising anti-cancer activity in zebrafish, activating the immune system to inhibit angiogenesis through toll-like receptor and vascular endothelial growth factor signaling pathways [43, 44]. Extracts of Fo mycelium neutralized influenza A viruses including H5N1 in vitro, reducing infective virus titers at concentrations that were not cytotoxic [45]. In addition, Fo demonstrated in vitro activity against HSV, H5N1, H3N2, and Orthopox viruses [45–48], and bacteria including *Staphylococcus aureus* and *Mycobacterium tuberculosis* [47, 49]. Further, Fo triterpenoids exhibited anti-inflammatory effects in RAW-264.7 murine macrophages [50]. 

Various polypore fungi, including Tv and Fo, demonstrate immunomodulatory effects, possibly through increased production of IL-1 receptor antagonist (IL-1Ra) [31], a known immunoregulatory mediator. Normally, differentiation of germinal-center (GC) B cells in draining lymph nodes to memory phenotypes occurs after IL-1 levels have reached their post-vaccination peak [51]. Fungi, by increasing IL-1Ra and attenuating IL-6/TNF-α [51], could thereby support GC function and subsequent memory formation. This process may occur directly (through inhibition of IL-1 signaling via IL-1Ra), and indirectly (through attenuation of IL-6 and TNF-α, thereby supporting GC function and differentiation of T follicular helper cells). Notably, Fo and Tv, evaluated in the current study in combination (FoTv) as an adjunct to COVID-19 vaccination, is being examined as a treatment for active COVID-19 infection in a randomized, placebo-controlled clinical trial of patients with mild-to-moderate illness [52, 53]. 

The primary objective of the current study was to examine effects of adjunctive short-term oral supplementation with FoTv versus Placebo to standard COVID-19 vaccination on vaccine reactogenicity and immunogenicity. We specifically hypothesized that: H1: Post-vaccination FoTv treatment would be (a) safe and (b) feasible.

Because we anticipated that FoTv’s impact on vaccine side effects and Ab levels would be most readily detected among participants without prior SARS-CoV-2 Ag exposure (from prior infection or vaccination), outcomes in participants with or without exposure were examined separately. This approach reflects the findings that (1) prior exposure to Ag from either SARS-CoV-2 infection or COVID-19 vaccination influences temporal dynamics of primary and memory Ab responses [21] and (2) repeated exposure to vaccine adjuvant increases severity of vaccine side effects [7, 8]. Therefore, we hypothesized that: H2: Individuals without prior SARS-CoV-2 Ag exposure who received FoTv would (a) exhibit fewer vaccine-related side effects across the treatment period relative to all other treatment/exposure subgroups (b) without adversely impacting their serum Ab levels.

Building on the primary objective of testing FoTv as a safe and feasible adjunct to COVID-19 vaccination, this study also explored the potential of mushroom mycelium supplementation to support broader immune protection against emerging SARS-CoV-2 variants and other infectious diseases, potentially serving as a tool to prevent infection from new strains of COVID-19 or mitigate future pandemics.

## Methods

### Study design

This was a 6-month, single-site, double-blind, placebo-controlled, randomized clinical trial. Participants were recruited from and consented at COVID-19 community vaccination sites in San Diego, California. Ethics approval was obtained from the University of California San Diego Institutional Review Board: 200633-1c. The trial protocol can be accessed at ClinicalTrials.gov, Identifier: NCT04951336 and this study adheres to CONSORT guidelines (see Supplemental Material 1).

### Participants

Participants consisted of 90 eligible individuals receiving COVID-19 vaccination in the San Diego area who provided written consent. Originally, a planned sample size of 66 was estimated based on the number needed to assess safety and feasibility. However, with additional funding, sample size was increased to 90 to permit assessment of efficacy outcomes and to better equalize the number of participants in each group (see Procedure). Computations were performed using the RMASS program by Hedeker [54] for a model with one between and one within factor (including up to 20% dropout rate) to provide minimum power of 80%. Sex was self-reported in response to an open-ended query.

### Eligibility criteria

*Inclusion*: (1) Scheduled to receive COVID-19 vaccination/booster; and (2) age ≥ 18.

*Exclusion*: (1) Currently using investigational agents to prevent/treat COVID-19 or immunosuppressive medication; (2) known renal/hepatic disease; (3) pregnant/lactating women; and (4) prisoners.

### Randomization and masking

**Fig. 1:**
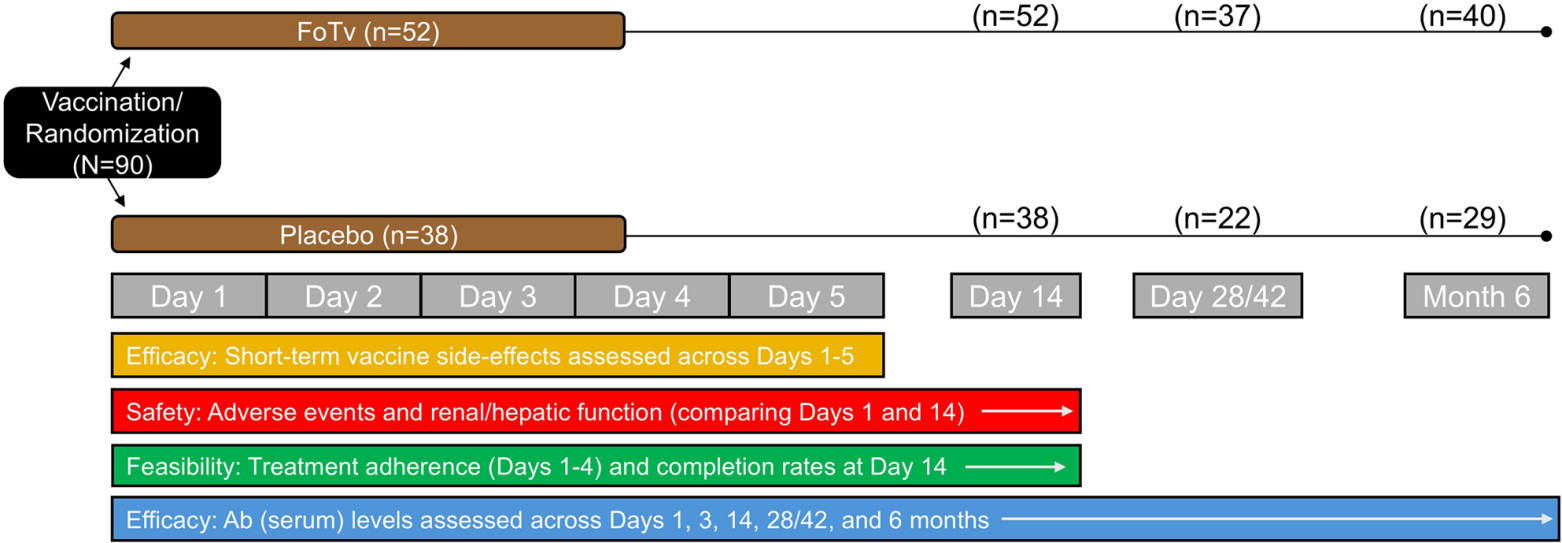
Design of Clinical Trial. At time of COVID-19 vaccination, participants were randomized to receive either a 4-day regimen of FoTv (*Fomitopsis officinalis/Trametes versicolor*) or Placebo on Day 1 (baseline). Vaccine side effects were assessed across Days 1–5. Adverse events were assessed across Days 1–14 and renal/hepatic function were assessed on Days 1 and 14. Feasibility analyses examined treatment adherence (Days 1–4) and completion rate at Day 14. Antibody (Ab) levels were assessed on Days 1, 3, 14, and 28/42, and at 6 months. For those who received a booster vaccine, Ab levels were obtained 3 days after a vaccine (Day 3). For those who received their first vaccine, Day 28/42 reflects a time point 28 days after vaccination for the one-dose vaccine, or 42 days after the first dose of the two-dose vaccine (i.e., 2 weeks after the second dose)

Participants were randomly assigned to receive FoTv or Placebo on the day of COVID-19 vaccination (day 1) (Fig. 1). Funding constraints dictated initial randomization to FoTv vs. Placebo groups in a 2:1 ratio, later adjusted to 1:1 after securing additional funding to facilitate equalizing the number of participants in each group and permit increased power for analyses of efficacy outcomes. Prior to recruitment, the statistician (S.G.) created a randomization table using block sizes of 4 and 6 (SPSS software). Only the statistician was unblinded to participant randomization. FoTv and Placebo capsules were visually identical and were distributed in unlabeled containers.

### Procedures

Short-term vaccine side effects were collected on days 1–5 (for side effect efficacy analyses) because side effects resolve in a few days [5]. To assess safety, blood from baseline and day 14 was used to measure renal/hepatic function, and adverse events were examined in this timeframe. To assess feasibility, medication adherence (Days 1–4) and completion rate at Day 14 were examined. To assess efficacy, SARS CoV-2 IgG Ab levels were obtained at the following timepoints: [1] single-dose vaccine (Johnson & Johnson) blood was collected on days 1, 14 (2 weeks after vaccination), and 28, and at 6-months post-vaccination; [2] two-dose vaccine (Pfizer-BioNTech/Moderna), on days 1, 14 (2 weeks after first dose of the two-dose vaccines), and 42 (two weeks after the second dose of the two-dose vaccines), and at 6-months post-vaccination; and [3] vaccine booster (Pfizer-BioNTech/Moderna), on days 1, 3, and 14 (2 weeks after booster vaccine), and at 6-months. The 6-month timepoint was chosen because, even with a 3–5 month half-life from peak titer, most individuals would still have measurable increases in Ab titer as a result of vaccination given at study onset [55].

FoTv consisted of freeze-dried FoTv mycelium fermented on a brown rice substrate, and inert Placebo of freeze-dried cooked organic brown rice. FoTv and Placebo were developed, encapsulated to a cGMP standard, and provided by Fungi Perfecti, LLC (Supplemental Materials 2). Capsules were packaged and appeared identical to untrained study participants.

FoTv/Placebo dosage was eight capsules (500 mg/capsule) three times daily (TID) for four consecutive days (starting day of first vaccination/booster). It was based on a prior study of effects of Tv on immune response [34] and was the regimen employed in a clinical trial of FoTv in patients with active COVID-19 infection [53]. A short-term, 4-day, dosing regimen was selected to: (1) minimize subject burden and maximize adherence; and (2) permit delivery of FoTv’s expected immunomodulatory effects when vaccine-expressed Ag is present and when vaccine-related, short-term side effects are greatest.

At end of enrollment, COVID Exposure Status was ascertained from baseline blood specimens. A Meso Scale Discovery, Multi-Spot Assay System / V-PLEX COVID-19 Serology Kit was used to obtain serum levels of Ab proteins at Days 1, 3 (vaccine booster only), 14, 28/42 (single [28] or two-dose [42] vaccine only), and 6 months. Those with detectable anti-SARS-CoV-2 Abs (from prior COVID-19 infection or vaccination) were classified as “COVID-Exposed” and those with undetectable anti-SARS-CoV-2 Abs (concentration of nucleocapsid [N] protein < 3 BAU [binding antibody units]/mL and concentration of receptor-binding domain (RBD) or Spike proteins < 4 BAU) as “COVID-Naive”. For the four participants missing Day 1 Ab data, COVID-Exposure Status stratum was assigned based on a self-report measure. For this measure, participants were asked to state whether they had previously had COVID-19 or had received a prior COVID vaccination, and if so, when. Note that all other participants in the COVID-Naïve group denied having prior vaccination or COVID-infection.

Serostatus thresholds (*N* < 3 BAU/mL; RBD or Spike < 4 BAU/mL) were selected a priori to be conservative relative to common manufacturer cut-offs after WHO BAU standardization [e.g., Roche Elecsys ≥ 0.8 BAU/mL indicates reactivity [56], thereby minimizing false positives in the ‘Naive’ group [57]. While we did not perform neutralization assays, literature shows binding Ab levels in the ~ 90–150 BAU/mL range correlate with neutralization and ~ 456 BAU/mL predicts substantial neutralizing capacity; our thresholds are intentionally well below such ranges [22, 58, 59]. We also observed large baseline separations between Naïve and Exposed groups (see Supplemental Materials 2, Supplemental Table 3).

### Outcomes

*Safety*: (1) Adverse event (hospitalizations; ER or urgent care visits) frequency across days 1–14; and (2) serum renal/hepatic function markers on days 1 and 14. Participants were classified as having normal vs. abnormal renal function using estimated glomerular filtration rate [60] (eGFR) and normal vs. abnormal hepatic function based on serum aspartate aminotransferase, alanine transaminase, and alkaline phosphatase. Cutoff scores for normal vs. abnormal classifications appear in Supplemental Materials 2, Supplemental Table 1.

*Feasibility*: (1) Completion rate at Day 14 (end of safety assessment period); and (2) Treatment Adherence= (capsules taken/assigned) * 100%.

*Efficacy*: (1) Duration and number of COVID-19 vaccine/booster side effects; and (2) Ab levels for RBD and Spike. Participants rated side effects as 0 (none or absent) to 4 (very severe) daily for Days 1–5. All CDC vaccine-associated side effects (5) were included: Feeling feverish, low-grade afternoon fever, alternating fever or chills, chills, fatigue, muscle aches, nausea, headaches, redness, injection site swelling, and injection site pain (Supplemental Materials 2, Supplemental Table 2). The primary side effect outcome was the total number of side effects present (Side Effect Count), which was summed for each day. A secondary and more subjective side effect outcome (Side Effect Severity), the sum of side effect severities for each day, was computed to explore whether FoTv reduced the severity of side effects.

### Data management and statistical analysis

Data was managed, and statistical analyses were performed, by Krupp Center for Integrative Research (KCIR) biostatistics and data management core, using the KCIR Data System. Each participant was assigned a sequential participant number. Baseline group demographic and clinical feature comparability was tested using Analysis of Variance and Chi-square analyses. Descriptive analyses assessed data distribution, normality, and homogeneity. Missing data and group dropout rate were examined for randomness [61]. Data were analyzed in SPSS (v.28) using two-tailed statistical tests (differences considered statistically significant for *p *-values < 0.05).

Safety analyses were based on percentage (95% CI) of participants transitioning from normal renal/hepatic function on Day 1 to abnormal on Day 14. Feasibility used mean and 95% CIs to ascertain whether 80% completion/adherence was attained. Efficacy results employed linear mixed-effects (LME) analyses. The first analysis compared Side Effect Count between two grouping factors, (1) Treatment Group (FoTv vs. Placebo) and (2) COVID Exposure Status (COVID-Naive vs. COVID-Exposed), across five time points (Days 1–5). The same model was employed to perform an exploratory analysis on Side Effect Severity. The second analysis compared Abs between the same grouping factors, across four time points (Days 3, 14, 28/42, and 6 months). Note that the Day 28 or Day 42 (28/42) timepoint depended on the type of vaccination and whether it required a single dose (Ab levels assessed on Day 28) or two doses (Ab levels assessed on Day 42). Data from Day 28 or 42 were combined into a single Day 28/42. Because Ab levels from Day 1 were used to create COVID-Exposure Status strata, longitudinal analyses excluded Day 1 data when comparing across strata.

## Results

Participants were enrolled between June 2021-January 2022, with the last follow-up visit in June 2022. Ninety participants were enrolled, 52 receiving FoTv and 38 receiving Placebo. Mean participant age was 39.0 ± 15.6 years. Participants were: 57.3% female; 74.2% Caucasian, 19.1% Asian, 3.4% African American, 3.4% other race; and 22.5% Hispanic. Because of 2:1 FoTv-to-Placebo randomization ratio early in the study, together with vaccine policies then in place prioritizing older individuals, the FoTv group had a larger proportion of older individuals. Beyond age, there were no significant differences between FoTv and Placebo groups in demographic/clinical characteristics (Table 1) or efficacy variables (side effects or Abs) at baseline (all *p*-values > 0.162). Accordingly, age was included as a covariate for analyses of continuous variables. Seventeen participants (19%) did not complete all study visits: Between days 14–28/42, one participant relocated, and another was lost to follow-up. Between days 28/42 − 6 months, 15 additional participants were lost to follow-up. Dropout rate was not significantly associated with Treatment Group (χ^2^ = 0.201, df = 1, *p* = 0.654). In addition, there were no significant differences at baseline between completers and non-completers with respect to either hepatic and renal function markers (*p* values > 0.457) or Ab or side-effect efficacy measures (*p*-values > 0.592). The same was true for demographic characteristics (*p*-values > 0.112), except for age where completers were significantly older (9 years older, *p* = 0.038) than non-completers. Importantly, there was not a significant interaction between dropout rate and Treatment Group (*p* = 0.956) for age. All participants were included Ab analyses regardless of if/when they dropped out.

**Table 1:** Patient Demographics and Baseline Clinical Characteristics

Characteristic	FoTv (*n* = 52)	Placebo (*n* = 38)	Statistic	df	*p* value	
	Mean/Count (SD/%)	Mean/Count (SD/%)	F or χ^2^			
Age	43.1 (15.6)	33.5 (13.9)	8.98	1,88	0.004	
Body Mass Index	27.4 (7.8)	25.3 (4.8)	2.20	1,87	0.142	
Smoker	10 (29.4%)	4 (19.0%)	0.735	1	0.391	
Sex			0.170	1	0.680	
Female	31 (59.6%)	21 (55.3%)				
Male	21 (40.4%)	17 (44.7%)				
Self-reported race			4.69	3	0.196	
Asian	9 (17.3%)	8 (21.1%)				
Caucasian	41 (78.8%)	26 (68.4%)				
African	2 (3.8%)	1 (2.6%)				
Other	0 (0%)	3 (7.8%)				
Self-reported ethnicity			0.327	1	0.567	
Hispanic	11 (21.2%)	10 (26.3%)				
Not Hispanic	41 (78.8%)	28 (73.7%)				
Vaccine Type			1.47	2	0.480	
Moderna	14 (26.9%)	13 (34.2%)				
Pfizer	28 (53.8%)	21 (55.3%)				
Johnson & Johnson	10 (19.2%)	4 (10.5%)				
Renal Function						
eGFR level	109.4 (26.0)	116.6 (20.2)	1.993	1,87	0.162	
Normal eGFR (%)	74.5%	92.1%	4.57	1	0.032	
Hepatic Function						
AST						
AST level	20.1 (7.4)	20.6 (5.8)	0.092	1,88	0.763	
Normal AST (%)	96.2%	100.0%	1.50	1	0.507*	
ALT						
ALT level	26.2 (21.5)	21.2 (12.9)	1.615	1,88	0.207	
Normal ALT (%)	82.7%	89.5%	0.82	1	0.366	
ALP						
ALP level	76.0 (21.0)	75.2 (25.5)	0.027	1,88	0.871	
Normal ALP (%)	100.0%	94.7%	2.78	1	0.176*	

F-values and Chi-square statistics are reported for continuous and categorical variables, respectively. *Due to the small sample size for cells in these analyses, probability values for Fischer’s Exact tests are reported in lieu of Chi-square tests. Renal and Hepatic Function percentages reflect the percentage of participants who were normal at baseline. *AST* Aspartate Aminotransferase (U/L), *ALT* Alanine Transaminase (U/L), *ALP* Alkaline Phosphatase (U/L), and *eGFR* adjusted glomerular filtration rate (mL/min). Smoking data (Never Smoker vs. Smoker [Current Daily, Current Some Days, Former Smoker, or Passive Smoker]), were not available for 18 participants in the FoTv group and 17 participants in the Placebo group

### Safety

No adverse events were reported in either FoTv or Placebo group. At baseline (and day 14, χ^2^ = 4.63, *p* = 0.032), only renal function (percent of participants with normal eGFR) was significantly higher in the Placebo group. However, this inherent baseline characteristic of participants randomized to FoTv (vs. Placebo) possibly reflected their older mean age (Table 1). Importantly, there were overlapping CIs for the percentage of participants in each Treatment Group transitioning from normal to abnormal renal or hepatic function across this timeframe (Table 2).

**Table 2:** Percentage of Participants Transitioning from Normal Renal or Hepatic Function at Baseline to Abnormal at Day 14

Day 1 versus Day 14:Percentage Transitioning from Normal to Abnormal Function				
	FoTv		Placebo	
	% Transitioning	95% CI	% Transitioning	95% CI
Renal Function				
Adjusted eGFR	10.5	2.9-24.8	2.9	0.1-14.9
Hepatic Function				
AST	2.0	0.1-10.6	2.6	0.1-13.8
ALT	4.7	0.6-16.2	5.9	0.7-19.7
ALP	1.9	0.0-10.4	0.0	0.0-10.6

The Normal to Abnormal transition analysis reflects the percentage of participants with normal function at Day 1 that transitioned to abnormal at Day 14 compared to participants with normal function at both Days, across the Treatment Groups. *AST *Aspartate Aminotransferase, *ALT *Alanine Transaminase, *ALP *Alkaline Phosphatase, and *eGFR* glomerular filtration rate

### Feasibility

**Fig. 2:**
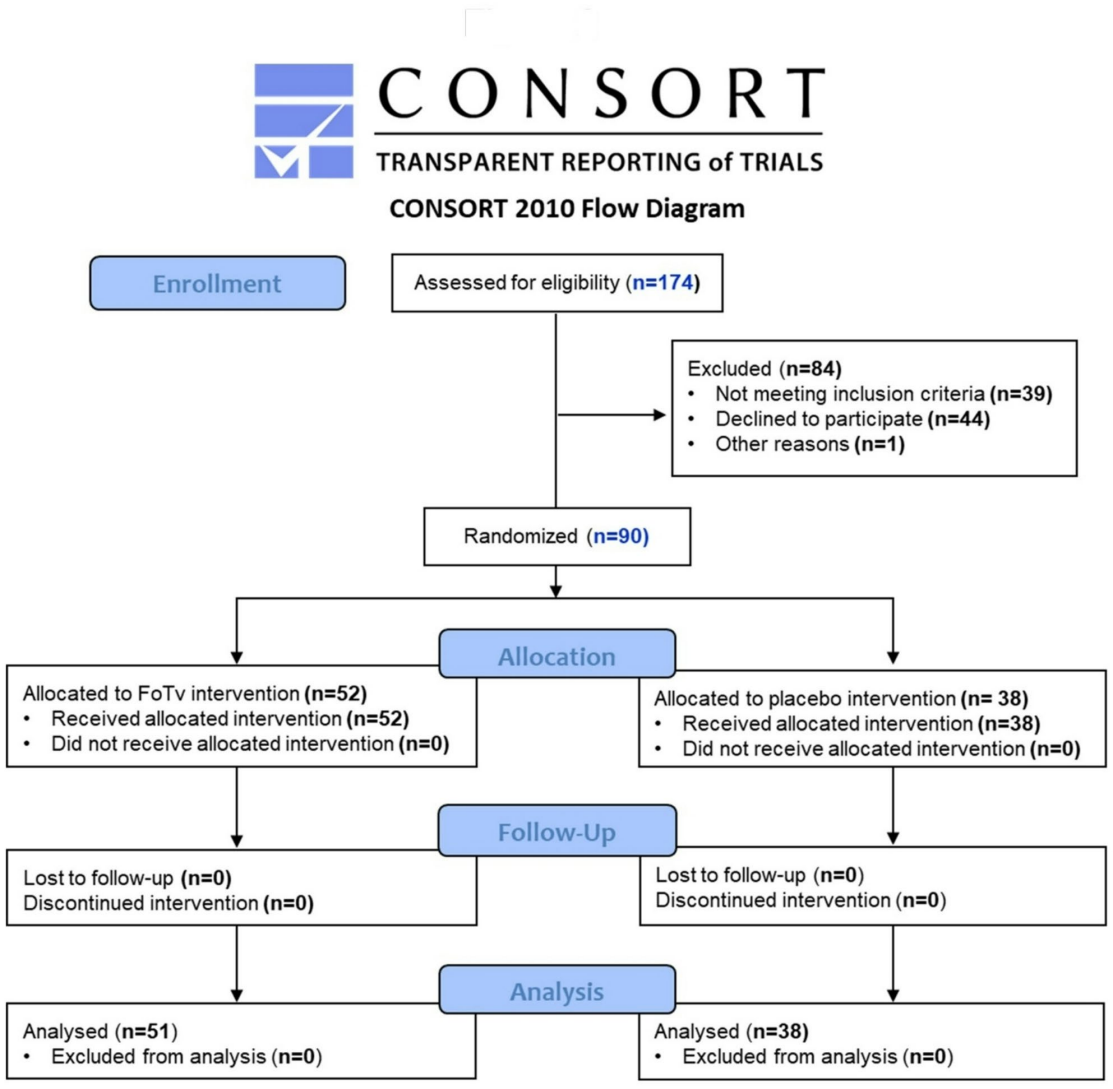
Consort Diagram for Clinical Trial Safety and Feasibility Data from Day 1 (baseline) to Day 14

Feasibility analyses examined eligibility for 174 participants, of whom 84 were excluded, and 90 were enrolled (Fig. 2). After enrollment, all participants (100%) completed days 1–14 of the study (Fig. 2). Treatment Adherence was high (96.0%±7.3%, CI: 94.4–97.5%); and was similar for FoTv (96.4%±6.7%, CI: 94.5–98.3%) and Placebo (95.4%±8.1%, CI: 92.7–98.1%).

### Efficacy

Prior to efficacy analyses, five participants (2 Placebo, 3 FoTv) who had *≥* 1 extreme outlier Ab level (> 3 SD above mean) were excluded. Two were in the COVID-Naive FoTv subgroup, two were in the COVID-Exposed Placebo subgroup, and one was in the COVID-Exposed Active subgroup. This resulted in the following groups: FoTv: COVID-Naive (*n* = 19), COVID-Exposed (*n* = 30); Placebo: COVID-Naive (*n* = 11), COVID-Exposed (*n* = 25). Note that three of these participants had contracted COVID-19 or received the vaccine booster prior to the 6-month time point, possibly contributing to their extreme data. Efficacy analyses examined Treatment Group by COVID-Exposure Status by Day interactions. Follow-up LME analyses were carried out separately for COVID-Exposure Status strata and Treatment Groups.

### Side effects

**Fig. 3:**
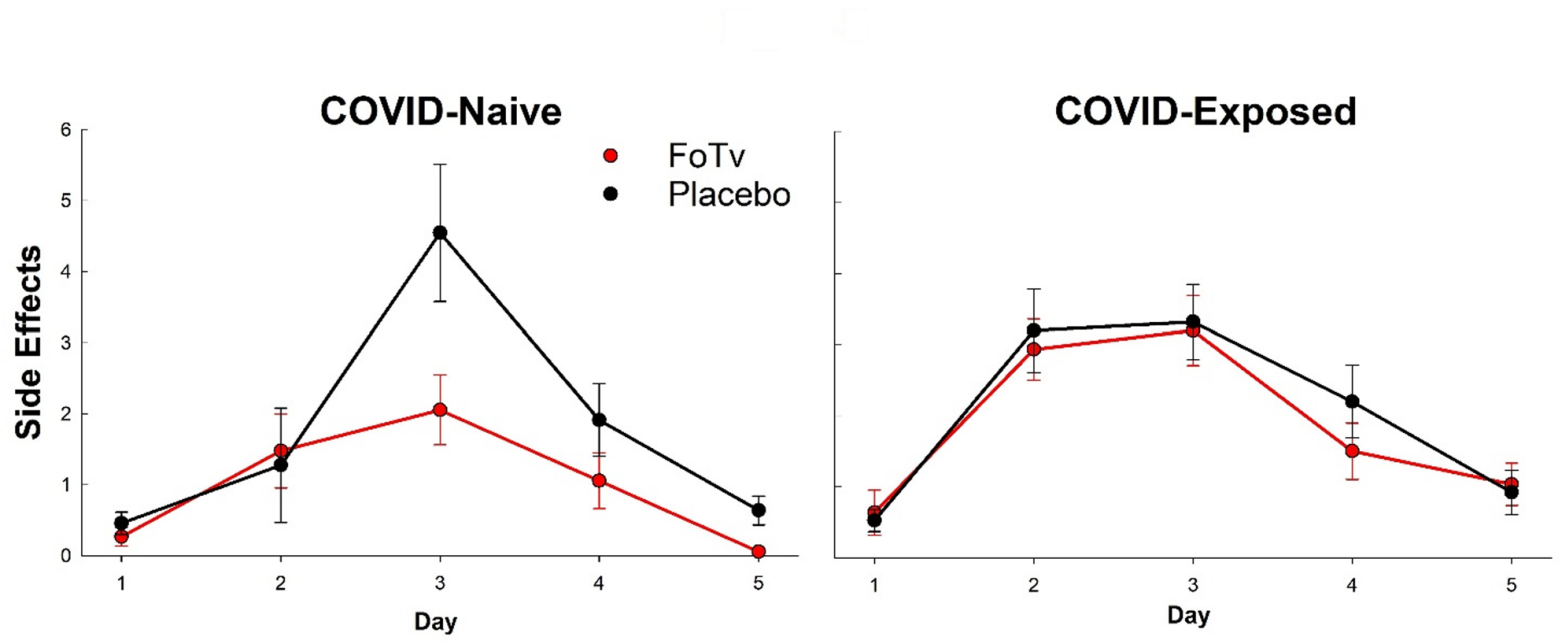
Efficacy results for Side Effects. Side Effect Counts are shown across days 1 to 5 for FoTv (red) and Placebo (black) groups who were COVID-Naive (left) or COVID-Exposed (right) prior to vaccine/booster. The COVID-Naive FoTv group (*n* = 19) exhibited significantly fewer side effects across days 1–5 relative to the Placebo group (*n* = 11), whereas the COVID-Exposed FoTv (*n* = 30) and Placebo (*n* = 25) groups did not. Error bars reflect SEM

*Primary Analysis: Side Effect Count.* Presentation of vaccine-related side effects (over the first 5 days post-vaccination) depended on both prior COVID exposure and FoTv versus Placebo assignment. There was a strong trend for a Treatment Group by COVID Exposure Status by Day interaction (Days 1–5) for Side effect Count (F_(8,325)_ = 1.898, *p* = 0.060; Fig. 3). This result was almost identical when dropout status was added to the model. The first set of follow up analyses was carried out separately for each COVID Exposure Status strata. There was a significant Treatment Group by Day interaction for COVID-Naive (F_(4,112)_ = 2.956, *p* = 0.023), but not COVID-Exposed (F_(4,212)_ = 0.436, *p* = 0.782), stratum. Furthermore, the FoTv Naive group reported significantly fewer side effects than Placebo on days 3 and 5 (F-values_(1,27)_ > 5.624, *p*-values < 0.026; Fig. 3), whereas the FoTv COVID-Exposed group was not significantly different from Placebo on any day (F-values_(1,52)_ < 0.679, *p*-values > 0.413)(Fig. 3).

*Exploratory Analysis: Side Effect Severity.* There was not a significant Treatment Group by COVID Exposure Status by Day interaction (Days 1–5) for Side effect Severity (F_(8,309)_ = 1.156, *p* = 0.326). This result was almost identical when dropout status was added to the model. There was a non-significant trend for a Treatment Group by Day interaction for COVID-Naive (F_(4,104)_ = 1.780, *p* = 0.138), but not COVID-Exposed (F_(4,204)_ = 0.551, *p* = 0.699), stratum. The FoTv group reported significantly fewer side effects than Placebo on day 5 (F_(1,27)_ = 8.008, *p*-values = 0.009) and a non-significant trend on day 3 (F_(1,27)_ = 3.225, *p* = 0.084), whereas the FoTv COVID-Exposed group was not significantly different from Placebo on any day (F-values_(1,52)_ < 1.074, *p*-values > 0.305).

### Antibodies

**Fig. 4:**
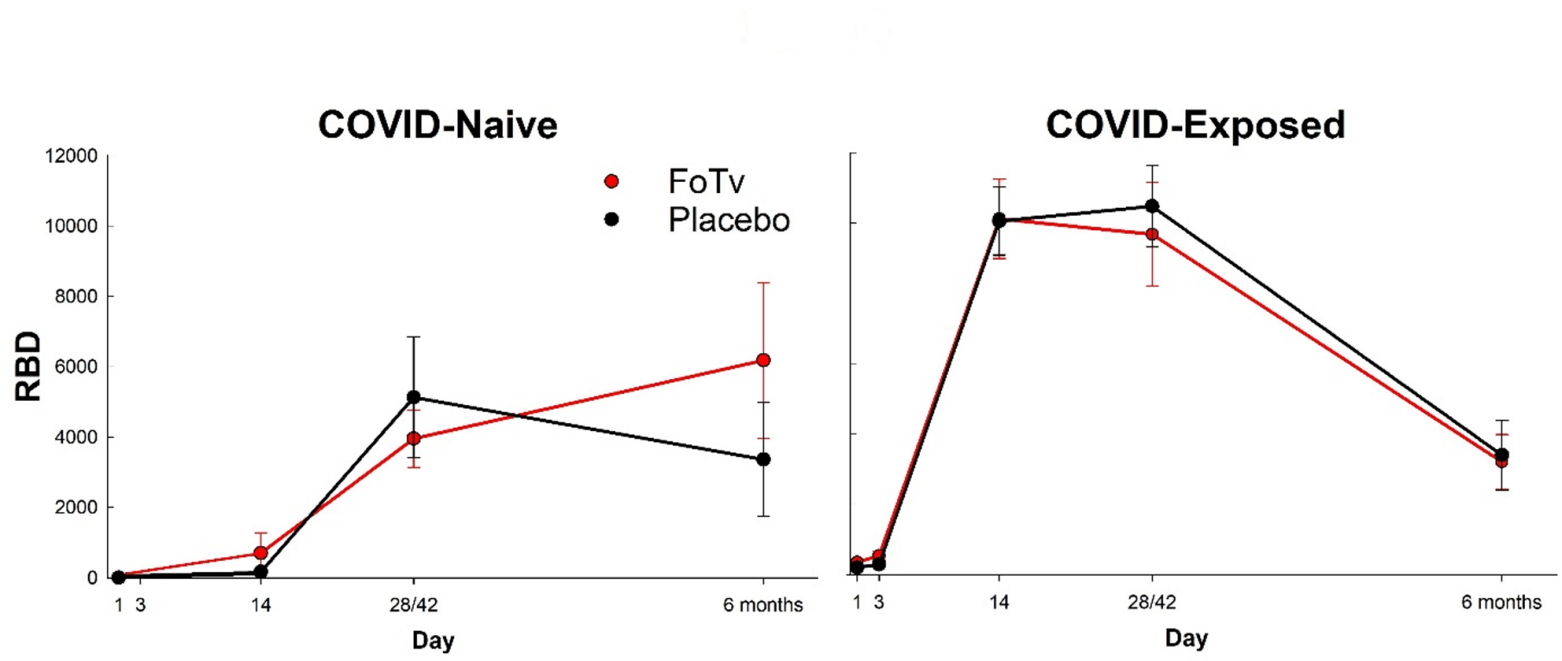
Efficacy results for antibodies. RBD antibody response across day 1 to 6 months for FoTv (red) and Placebo (black) groups in participants who were COVID-Naive (left) or COVID-Exposed (right) prior to vaccine/booster. The COVID-Naive FoTv group (*n* = 19) exhibited significant increases from day 14 to 6 months, while the COVID-Exposed FoTv group (*n* = 30) exhibited significant decreases over this period (see follow up t-tests in Results section). By contrast, the COVID-Naive (*n* = 11) and COVID-Exposed (*n* = 25) RBD antibody response across days in the Placebo groups did not significantly differ from each other (see follow up t-tests in Results section). Error bars reflect SEM. RBD (anti-SARS-CoV-2 receptor binding domain) units are BAU (binding antibody units)/mL

Ab levels (across 6 months) also depended on both prior COVID exposure and FoTv vs. Placebo assignment. There was a significant Treatment Group by COVID Exposure Status by Day (3, 14, 28/42, and 6 months) interaction for RBD (F_(2,150)_ = 6.374, *p* = 0.002; Fig. 4) and Spike: (F_(2,157)_ = 4.286, *p* = 0.002) Abs. Within each COVID Exposure Status stratum, there were no significant Treatment Group by Day interactions (all F-values_(1,53−144)_ < 1.080, all *p*-values > 0.303). Within Treatment Groups, there were significant COVID Exposure Status by Day interactions for FoTv (RBD: F_(1,87)_ = 12.612, *p* < 0.001; Spike: F_(1,91)_ = 8.358, *p* = 0.005), but not Placebo (RBD: F_(1,65)_ = 1.379, *p* = 0.245; Spike: F_(1,65)_ = 1.170, *p* = 0.283). Follow-up, pairwise comparisons of Ab levels across Days detected a more durable Ab response in the COVID-Naive FoTv group, which exhibited significant RBD increases across timepoints (Day 14–28/42; Day 14 − 6 months; all t-values > 2.314, df = 31–33, all *p-*values < 0.027), whereas the COVID-Exposed FoTv group exhibited significant decreases (Day 14 − 6 months; t = -8.561, df = 39, *p* < 0.001). A pattern similar to RBD was observed for Spike Ab. In the COVID-Naive FoTv group there was a significant increase across timepoints (Day 14–28/42; Day 14 − 6 months; all t-values > 3.527, df = 31–32, all *p*-values < 0.001), suggesting a more durable Ab response. However, there were significant decreases for the COVID-Exposed FoTv group (Day 14 − 6 months; (t=-11.996, df = 39, *p* < 0.001). The strength of the FoTv effect on Ab levels was even stronger when the five outlier participants with extremely high Ab levels were included in the analysis. The main findings were almost identical when missing data were imputed using a fully conditional specification multiple imputation method with five imputations.

## Discussion

This study addressed whether FoTv could serve as a safe, feasible, and effective adjunct to COVID-19 vaccination. Short-term (4-day) oral FoTv administration was safe (Table 2) and feasible (Fig. 2). In those who had not been exposed to COVID-19 infection or received prior vaccination (i.e., COVID-Naive individuals), FoTv also reduced the number of short-term (5-day period post-vaccination) side effects (Fig. 3), and possibly side effect severity, while preventing the normal decay of vaccine Ab responses at 6 months (end of study; Fig. 4). If anything, FoTv may have *increased* Ab response durability, in that the COVID-Naive FoTv group exhibited increasing Ab levels from Day 14 onward, whereas all other groups did not. FoTv constituted a safe, acceptable adjunct to vaccination for COVID-19 and, an optimal characteristic vaccine adjuvant or adjunct, it may have decreased reactogenicity while preserving or increasing immunogenicity.

This pattern, minimized short-term side effects coupled with increased Ab durability, suggests immunomodulation, and is consistent with what is known about the immunobiology of fungi. Despite earlier speculation that fungal polysaccharides might stimulate excessive reactogenicity and potentially trigger cytokine storm during acute COVID-19 infection [62], a recent clinical study of COVID-19 patients instead found fungal β-glucan treatment decreased inflammatory markers (IL-6 and d-Dimer) [63]. Further, this idea is reinforced by the fact that polypore fungi contain various bioactive peptides [64], compounds known to regulate cytokine responses required for an optimal adaptive immune response [65]. 

In the COVID-Naive stratum, reduced reactogenicity is consistent with an anti-inflammatory effect [66] of FoTv. FoTv also produced preserved or more gradual Ab trajectories and ultimately numerically higher peak Ab responses than Placebo (Fig. 3B), suggesting it may have facilitated a well-regulated immune response and led to sustained, long-lasting immunity [67–71]. Preserved antibody durability is consistent with a regulatory effect marked by memory B/T-cell formation. However, because we did not obtain objective measures of inflammation (e.g., IL-1/IL-1Ra, IL-6, TNF-α) or memory formation (e.g., B/T-cell phenotypes, Tfh/GC metrics), confirmation of this suggested mechanism would require future studies employing direct assessment of such outcome measures.

It is also worth considering that the effects of FoTv on Ab levels may not be solely B-cell mediated. In a Phase I clinical trial in women with breast cancer, daily oral administration of Tv (a component of FoTv) for 6 weeks elicited a dose-dependent increase in CD8 + T-cell counts [34]. This clinical trial suggests that an augmentative effect of Tv on vaccination may occur, at least in part, via its effect on CD8 + T-cells [72]. Further, lack of Ab decay may suggest a more gradual immune response and mitigation of vaccine-triggered overproduction of acute-phase inflammatory mediators.

Regardless of the mechanism, a natural adjunct that does not compromise (and potentially increases) Ab response durability could reduce vaccine hesitancy due to lessened need for/frequency of boosters. Moreover, an adjunct that reduces vaccine-related side effects could further reduce vaccine hesitancy. Such an adjunct would be highly desirable both for future COVID-19 strains and other emerging infectious diseases for which vaccination (e.g., mRNA vaccines) will likely constitute an important part of the collective strategy. Our findings provide support for the possible use of FoTv as an adjunct to primary vaccination. Because we did not measure outcomes other than Ab levels and side effects, it remains uncertain whether FoTv may offer benefit as an adjunct for booster vaccines. Given that FoTv appears to be a safe adjunct to vaccination, further study would be helpful to clarify the specificity of the findings.

A critical problematic feature of COVID-19 has been its continuous spawning of new, mutant strains with potentially greater transmissibility or lethality than the parent strain. Such viral mutability is also likely to be characteristic of other emerging infectious diseases (e.g., H5N1). Like Ichinohe et al., we found a fungal adjunct to vaccination positively impacted Ab levels. Ichinohe et al. additionally found that intranasal coadministration of fungal adjuvant with H5N1 vaccination not only boosted antibody production but also induced cross-protection (i.e., without requiring specific pathogen recognition) against both homologous and heterologous H5N1 strains [27]. They also found that this effect occurred because of mechanisms that were both MyD88-dependent (e.g., mediated by Toll-like-receptors) and MyD88-independent (e.g., mediated by dectin-1 receptors [which bind fungal polysaccharides]). The latter finding raises the prospect that fungal mycelia may have broadly activated multiple innate immune pathways and may serve as universal immune modulators that could strengthen overall host defense systems. Our results, taken together with those of Ichinohe et al., suggest that use of a fungal mycelial adjunct to vaccination may not only increase Ab levels, but might also be predicated upon mechanisms that could generalize to future vaccines for emerging infectious diseases and to mutant strains of these viruses as well.

Fungal mycelial products may be practical for widespread, standardized use as vaccine adjuncts/adjuvants for a number of reasons: (1) because fungi are chemically complex and biosynthesize numerous compounds, they may work synergistically to address several aspects of innate and acquired immunity; (2) they may constitute a safe, natural, sustainable, scalable, low-cost, and potentially immunomodulatory strategy to reduce side effects, while preserving (and possibly increasing) Ab responses; and (3) the technology that employs aseptic cultivation and solid-state fermentation already exists, allowing for safe, rapid, and high-volume production. In addition to the potential for mushroom mycelium to produce unique tissue-specific compounds [73], large-scale medical use of mushroom fruit bodies may not be feasible due to impracticality of controlled cultivation, concerns about their overharvesting [74], and potential for contamination with algae/other microorganisms [75]. Conversely, the use of cultivated mycelia as a natural vaccine adjunct constitutes a realizable, standardizable, and scalable strategy for population-level pandemic preparedness.

This study had several limitations warranting discussion. Because of rapidly changing nature of the COVID-19 pandemic, the protocol was unable to begin recruitment until after winter 2020/2021 COVID-19 peak (after initial vaccine rollout). Earlier launch might have resulted in a larger proportion of COVID-Naive participants. Also, a minority of previously vaccinated participants with profound titer waning could have theoretically fallen below our BAU thresholds and been misclassified as COVID-Naive. Baseline BAU values in our cohort, however, showed strong separation (Supplemental Materials 2, Supplemental Table 3), suggesting that such misclassification—had it been present—would have been rare [76–78]. If so, this would have biased findings toward the null hypothesis. An additional limitation is that we were not able to conduct a viral neutralization assay that would have been needed to confirm that Abs were indeed neutralizing the virus. Rather, the protocol was designed specifically to measure binding Abs to Spike, RBD, and N. These assays are widely used in SARS-CoV-2 vaccine studies and are known correlates of neutralizing activity at the population level, though they do not directly confirm functional neutralization. It was also not logistically feasible to determine incidence of SARS-CoV-2 infection post-vaccination; biomarkers of infection risk (RBD/Spike Ab levels) were employed instead. Moreover, because Ab levels beyond six months were not examined, the extent of FoTv’s long-term effects may not have been fully appreciated (Fig. 3). Additionally, other markers (e.g., inflammatory cytokine, memory B- and T-cells) that could have better elucidated the nature, or possible broadening, of the immune response were not examined. Attrition bias may have influenced the Ab results because completers and non-completers differed with respect to age. However, neither the Treatment Groups nor the COVID-Exposure Status strata differed in dropout rate, and age was included as a covariate in our Ab models. Finally, it was not feasible to examine a variety of other dosage/timing regimens. Similarly, our FoTv safety findings may not generalize to long-term use or more broadly to individuals within specific vulnerable subpopulations that were either enrolled in insufficient numbers in, or excluded from, this study. Interpretation of FoTv safety is also limited by the fact that we did not obtain serum for safety analysis until Day 14 (i.e., when Abs were examined) as a means to minimize venipuncture visits and patient burden. This may have increased the possibility that we may have failed to detect a transiently abnormal finding which then normalized by Day 14.

## Conclusions

This is, to our knowledge, the first placebo-controlled human clinical trial of a fungal-based natural product as an adjunct to human vaccination and, more specifically, the first one to employ fungi in conjunction with mRNA-based or other vaccines for COVID-19. This early phase clinical trial demonstrated the safety and feasibility and provided a preliminary estimate of efficacy (short-term side effects/Ab responses), of FoTv as an adjunct to COVID-19 vaccination. In the process, it addressed and provided possible proof of the concept that in those receiving primary vaccination, fungi may simultaneously reduce vaccine reactogenicity without compromising (and possibly while increasing) immunogenicity. COVID-19 afforded the opportunities to: (a) test the strategy of using fungi as an adjunct to vaccination, and (b) do so during a pandemic caused by a novel virus.

More research is needed to elucidate precise mechanisms of fungal immunomodulation and specific clinical applications of fungi. However, the basic approach of using fungi as vaccine adjuncts appears to be safe and feasible, can be brought to bear rapidly, and may work with both mRNA and non-mRNA vaccines as well as with vaccines for other diseases [27]. For example, because Fo mycelium has documented antiviral activity against H5N1 [45, 48], FoTv might also serve as a candidate therapeutic for H5N1. Like SARS-CoV-2 when it first appeared, H5N1 is a virus to which the human population is largely immunologically naive, and which could present suddenly and globally. These considerations take on even greater significance given the potential harm and disruption that could be posed by emerging viruses and infectious diseases.

## Supplementary Information

Supplementary Material 1.

Supplementary Material 2.

## Data Availability

Data are available upon request from the corresponding author.
