# Supplementary material for: Polypore mushroom mycelia as an adjunct to COVID-19 vaccination: a randomized clinical trial
Source: BMC Immunol. 2026 Jan 31;27:24. doi: 10.1186/s12865-026-00809-9 (PMC12955250; doi:10.1186/s12865-026-00809-9)
Supplement: Supplementary file 2 — Supplementary Material 2. [file 12865_2026_809_MOESM2_ESM.pdf]

Supplemental Materials

FoTv Formulation

Background

Early in the COVID-19 pandemic, the authors were interested in the potential of mycelium-based products to ameliorate COVID-19 clinical symptoms; later, interest grew in the potential of mushroom mycelium and fermented substrates to mitigate adverse effects and enhance efficacy of mRNA-based SARS-CoV2 vaccines. Two polypore mushroom species, Agarikon (*Fomitopsis officinalis*, Fo) and Turkey Tail (*Trametes versicolor*, Tv), were selected based on preclinical and clinical immunological and antiviral data. The FoTv formula consists of equal components of Fo and Tv, described below.

Materials & Methods

Fo and Tv mycelia were independently cultivated through solid state fermentation on an organic brown rice substrate. Each myceliated fermented substrate ingredient was frozen, dried, and milled into a powder before being combined evenly and encapsulated in 500-mg pullulan capsules to produce the FoTv formulation, which was granted Investigational New Drug (IND) status by the Food and Drug Administration (FDA). An unfermented brown rice substrate, processed in the same manner, was employed as the placebo in this clinical trial.

Supplemental Table 1. Cut off scores for identifying normal and abnormal values for liver and renal function

	Normal	Abnormal
Liver Function		
Aspartate Aminotransferase (AST)	≤ 40 U/L	> 40 U/L
Alanine Transaminase (ALT)	≤ 41 U/L	> 41 U/L
Alkaline Phosphatase (ALP)	≤ 129 U/L	> 129 U/L
Renal Function		
Adjusted glomerular filtration rate (Adj. eGFR)	> 60 mL/min	≤ 60 mL/min

Note. Adj. eGFR = 142 X minimum (standardized serum creatinine/K OR 1)$^{\alpha}$ X maximum(standardized serum creatinine/K OR 1)$^{-1.200}$ X 0.9938^{Age} X 1.012 [if female]. Serum creatinine = mg/dL. K = 0.7 (females) or 0.9 (males). α = -0.241 (females) or -0.302 (males). Equation based on National Kidney Foundation guidelines (https://www.kidney.org/ckd-epi-creatinine-equation-2021-0).

Supplemental Table 2. Treatment Group by Day Interaction Effects for Vaccine Side effects and Other Side effects

	COVID-Naive Group		COVID-Exposed Group	
Side effect	F value	P value	F value	P value
Vaccination Side effects				
Feeling feverish	2.055	0.092	0.972	0.424
Low fever in the afternoon	0.897	0.468	0.651	0.627
Alternating fever and chills	1.063	0.378	0.842	0.500
Chills	1.845	0.124	0.249	0.910
Fatigue	1.767	0.141	1.287	0.276
Muscle aches	1.132	0.346	0.852	0.494
Nausea	2.918	0.025	1.190	0.316
Headaches	2.009	0.099	0.645	0.631
Redness/swelling at injection site	0.105	0.981	0.473	0.755
Pain at injection site	0.932	0.449	0.475	0.754
Side effect Count	2.956	0.023	0.436	0.782
Side effect Severity	1.780	0.138	0.551	0.699
Other Side effects				
Belly bloat	0.893	0.471	0.464	0.762
Bitter taste	0.489	0.744	0.795	0.529
Chest fullness	1.555	0.192	1.210	0.308
Cold limbs	2.270	0.065	1.916	0.109
Diarrhea	2.458	0.050	0.546	0.702
Dizziness	2.709	0.034	1.121	0.348
Dry cough	1.248	0.295	0.968	0.426
Excess sweating	0.458	0.766	1.390	0.239
Excessive thirst	1.283	0.281	0.435	0.783
Heartbeat	1.177	0.324	1.686	0.155
Insomnia	0.863	0.489	1.253	0.290
Loose stools	0.492	0.741	1.543	0.191
Loss appetite	0.365	0.833	0.811	0.519
Nervousness	1.199	0.316	0.375	0.826
Restlessness	3.907	0.005	1.055	0.380
Runny nose	0.649	0.629	1.417	0.230
Shortness breath	2.162	0.078	1.047	0.384
Shortness breath exertion	0.000	1.000	0.403	0.806
Skin rashes	1.616	0.175	1.809	0.128
Sore throat	0.430	0.786	1.630	0.168
Stuffy nose	1.141	0.342	0.875	0.480
Swelling	1.790	0.137	0.400	0.809

Note. The degrees of freedom for the COVID-Naive comparisons were 4, 106 and for the COVID-Exposed comparisons were 4, 202.

Supplemental Table 3. Baseline Antibody Descriptive Information for COVID-Exposure Strata.

Antibody Concentration	COVID Naive N=32	COVID Exposed N=58
N		
Mean	0.557	17.876
Median	0.234	3.680
Standard Deviation	0.755	42.835
Interquartile Range	0.648	17.864
RBD		
Mean	1.256	301.612
Median	0.537	195.325
Standard Deviation	2.053	317.666
Interquartile Range	1.211	357.546
Spike		
Mean	0.585	212.612
Median	0.421	185.624
Standard Deviation	0.678	223.165
Interquartile Range	0.465	203.577

Note. At end of enrollment, COVID Exposure Status was ascertained from baseline blood specimens. Those with detectable anti-SARS-CoV-2 Abs (from prior COVID-19 infection or vaccination) were classified as "COVID-Exposed" and those with undetectable anti-SARS-CoV-2 Abs (concentration of nucleocapsid [N] protein <3 BAU [binding antibody units]/mL and concentration of receptor-binding domain (RBD) or Spike proteins <4 BAU) as "COVID-Naive". The four participants with missing Antibody data at Baseline (two COVID-Naive FoTv and two COVID-Exposed Placebo), were categorized into these COVID Exposure Status strata according to self-reported exposure status, and their baseline data were replaced with the mean of their respective COVID-Exposure Stratum for each Ab.

Supplemental Table 4. Descriptive Statistics for Side effect and Antibody Measures

	FoTv		Placebo	
	COVID Naive (n=19)	COVID Exposed (n=30)	COVID Naive (n=11)	COVID Exposed (n=25)
Side effects				
Count				
Day 1	0.24 (0.54)	0.65 (1.70)	0.45 (0.52)	0.48 (0.75)
Day 2	1.38 (2.18)	2.87 (2.35)	1.27 (2.69)	3.19 (2.83)
Day 3	2.10 (2.19)	3.13 (2.69)	4.55 (3.21)	3.26 (2.63)
Day 4	1.00 (1.64)	1.45 (2.16)	1.91 (1.70)	2.04 (2.53)
Day 5	0.10 (0.30)	1.00 (1.61)	0.64 (0.67)	0.85 (1.51)
Severity				
Day 1	0.24 (0.54)	1.06 (3.13)	0.45 (0.52)	0.63 (1.11)
Day 2	3.06 (5.27)	5.58 (4.49)	4.00 (7.13)	6.78 (6.80)
Day 3	3.52 (5.11)	6.00 (7.21)	8.36 (8.88)	5.41 (5.29)
Day 4	1.24 (2.28)	2.26 (4.34)	2.55 (2.84)	3.30 (5.36)
Day 5	0.10 (0.30)	1.35 (2.47)	0.91 (1.22)	1.15 (2.54)
Antibody				
RBD				
Day 1	1.3 (1.9)	355.0 (358.1)	1.4 (2.5)	206.2 (188.9)
Day 3	N/A	541.9 (456.8)	N/A	289.8 (217.4)
Day 14	700.6 (2530.5)	10116.1 (6190.4)	168.2 (162.0)	10053.0 (4834.7)
Day 28/42	3953.5 (3460.1)	9675.0 (5860.4)	5129.0 (5704.4)	10469.3 (3844.3)
6 months	6178.4 (8273.7)	3209.5 (3720.2)	3359.3 (5108)	3398.5 (4093.5)
Spike				
Day 1	0.5 (0.4)	247.6 (258.5)	0.8 (1.0)	149.4 (120.0)
Day 3	N/A	380.7 (307.5)	N/A	205.4 (143.3)
Day 14	392.5 (1047.7)	4488.1 (1542.1)	224.9 (242.1)	4877.9 (1549.0)
Day 28/42	1958.7 (1542.2)	3981.3 (1366.7)	2383.3 (2275.4)	4719.5 (1192.6)
6 months	2107.2 (2696.4)	1544.4 (1341.9)	1400.1 (1673.3)	1546.5 (1347.9)

Note. Means and SDs are reported. Antibodies are in BAU (binding antibody units)/mL. Day 3 measure of Ab was obtained only for those who received a vaccine booster (and who were previously exposed via prior vaccination).
